# Characterization and health risk assessment of airborne microplastics in Delhi NCR

**DOI:** 10.1038/s41598-025-04306-8

**Published:** 2025-07-15

**Authors:** Molla Nageswar Rao, Sachin D. Ghude, Sandip D. Nivdange, Rajani Panchang, Atar Singh Pipal, Arkabanee Mukherjee, Hardeep Sharma, Vikash Kumar

**Affiliations:** 1 Ministry of Earth Sciences, Indian Institute of Tropical Meteorology, Pune, 411008 India; 2 Department of Environmental Sciences, Savitribai Phule Pune University, Pune, 411007 India

**Keywords:** Microplastics, Air pollution, Health risk, Delhi, Atmospheric chemistry, Environmental sciences

## Abstract

Exposure to airborne microplastics (MPs) is an emerging environmental and public health concern due to their widespread presence and potential toxicity. This study presents the first comprehensive characterization of airborne MPs in Delhi, India, focusing on their seasonal distribution, morphology, chemical composition, and associated health risks. Particulate matter (PM₁₀, PM₂.₅, and PM₁) was collected using active samplers at Lodhi Road during winter (January–March) and summer (April–June) of 2024. Average(± SD) concentrations of MPs were 1.87 ± 0.5 MPs/m^3^ for PM₁₀, 0.51 ± 0.2 MPs/m^3^ for PM₂.₅, and 0.49 ± 0.2 MPs/m^3^ for PM₁, with notable seasonal variation. Fragments (66%) and fibers (32%) were the dominant morphologies, primarily white/transparent and blue in color, ranging from 1–1000 µm in size. Weathering features were observed across particles, chemical analysis identified polyethylene terephthalate (PET) and polyethylene (PE) as the predominant polymers, with trace elements like zinc (Zn), silicon (Si) and aluminium (Al) adsorbed onto their surfaces. Wind rose analysis indicated predominant MP transport from the northwest, highlighting regional source influence. Considering their capacity to carry harmful pollutants, MPs may pose inhalation related health risks. This study underscores the need to integrate MPs into air quality frameworks and advocates for long-term, multi-site monitoring to evaluate their broader public health impacts.

## Introduction

Plastics have become an integral part of human life, and over the past decade, global plastic production has surged by approximately 50%^1^. By 2021, production reached around 400 million tons^2^. If current trends continue, the volume of mismanaged plastic waste is projected to increase significantly, from approximately 100 million tons in 2015 to around 265 million tons by 2060^3^. Plastic pollution has emerged as one of the most pressing environmental challenges of our time, primarily because the production and consumption of plastics far exceed the capacity for their effective disposal, reuse, or recycling^4,5^. Some plastics can persist in the environment for up to 50 years, contributing to long-term pollution and environmental degradation^6^. In this context, MPs pollution is emerging as a growing concern, as plastic waste breaks down into tiny fragments and disperses into the environment^7–9^. MPs are generally classified into two types based on their origin: primary and secondary. Primary MPs are small plastic beads intentionally released into the environment, often found in products like plastic items and personal care products^10^. Secondary MPs, on the other hand, result from the breakdown of larger plastic objects due to factors such as sunlight, chemical reactions, or environmental wear and tear^11–13^.

MPs come in various shapes, including fragments, fibers, and films, with sizes ranging from less than 1 µm to 5 mm^14^. They are observed in a variety of colors, such as clear, blue, red, green, and yellow, and have been widely identified in ambient air^15–19^. The surface morphology of MPs can vary significantly depending on the type of polymer and the degree of environmental weathering, such as photodegradation, oxidation, or mechanical abrasion^20^. Chemically, MPs are most composed of synthetic polymers such as polyethylene (PE), polypropylene (PP), polystyrene (PS), polyethylene terephthalate (PET), polyamide (PA), and polyvinyl chloride (PVC)^21^^.^ These polymers are often embedded with chemical additives such as plasticizers, flame retardants, UV stabilizers, and pigments, which enhance their mechanical and functional properties. Furthermore, environmental exposure enables MPs to adsorb pollutants such as heavy metals, and persistent organic pollutants (POPs) onto their surfaces. As such, MPs act as carriers for both inherent and adsorbed hazardous substances. Their persistence, lightweight nature, and widespread anthropogenic use facilitate their accumulation in the environment, including in air, water, soil, and even food and beverages^22–27^. This complex chemical composition, combined with their ability to penetrate biological systems, raises serious concerns about their potential health effects particularly through inhalation, such as respiratory inflammation, oxidative stress, and possibly cancer^27–30^. Exposure to harmful airborne MPs on human health has concerned scientific attention in recent years^6,20,29,31,32^.

Microplastic particles in the air are increasingly being detected in various locations, particularly in urban areas and major cities such as Paris, London, and Dongguan^23,26,33^. According to the World Health Organization (WHO), half of the world’s 20 most polluted cities are in South Asia. The region’s high ambient particulate matter (PM) levels may enhance the atmospheric transport and deposition of MPs, particularly through interactions between MPs and airborne particles, which is a major environmental and health concern. MPs have the potential to remain suspended in the atmosphere for extended periods, significantly increasing the likelihood of human inhalation. Despite this, their health impacts remain poorly understood. India, as one of the fastest-developing countries in South Asia, faced significant plastic consumption during 2018–2019, with an estimated 18.5 million tonnes of plastic being used. Notably, 60% of this plastic (approximately 11 million tonnes) consisted of single use plastics, predominantly used for packaging (CPCB, 2022). The Central Pollution Control Board (CPCB) reports that India generates a staggering 25,940 tonnes of plastic waste per day, with Delhi alone contributing 689.8 tonnes daily the highest amount among India’s megacities^34^. This underscores the critical need to investigate airborne MP particles in urban settings, particularly in megacities like Delhi, to better understand potential human exposure to these pollutants. Assessing airborne MPs can yield vital insights into not only pollution levels but also the types, characteristics, and sources of these particles. Such data is essential for identifying pollution sources and evaluating the associated environmental and health risks. While plastic pollution is increasingly becoming a global concern, especially in rapidly developing nations, there is currently a significant research gap regarding airborne MPs in India, particularly in urban environments such as Delhi. Given the growing scale of plastic pollution and its persistence in the environment, investigating the movement and behavior of MPs in densely populated urban areas is of paramount importance. This study aims to fill this knowledge gap by providing a comprehensive analysis of the occurrence, characteristics, and potential health risks posed by atmospheric MPs in Delhi.

**Fig. 1:**
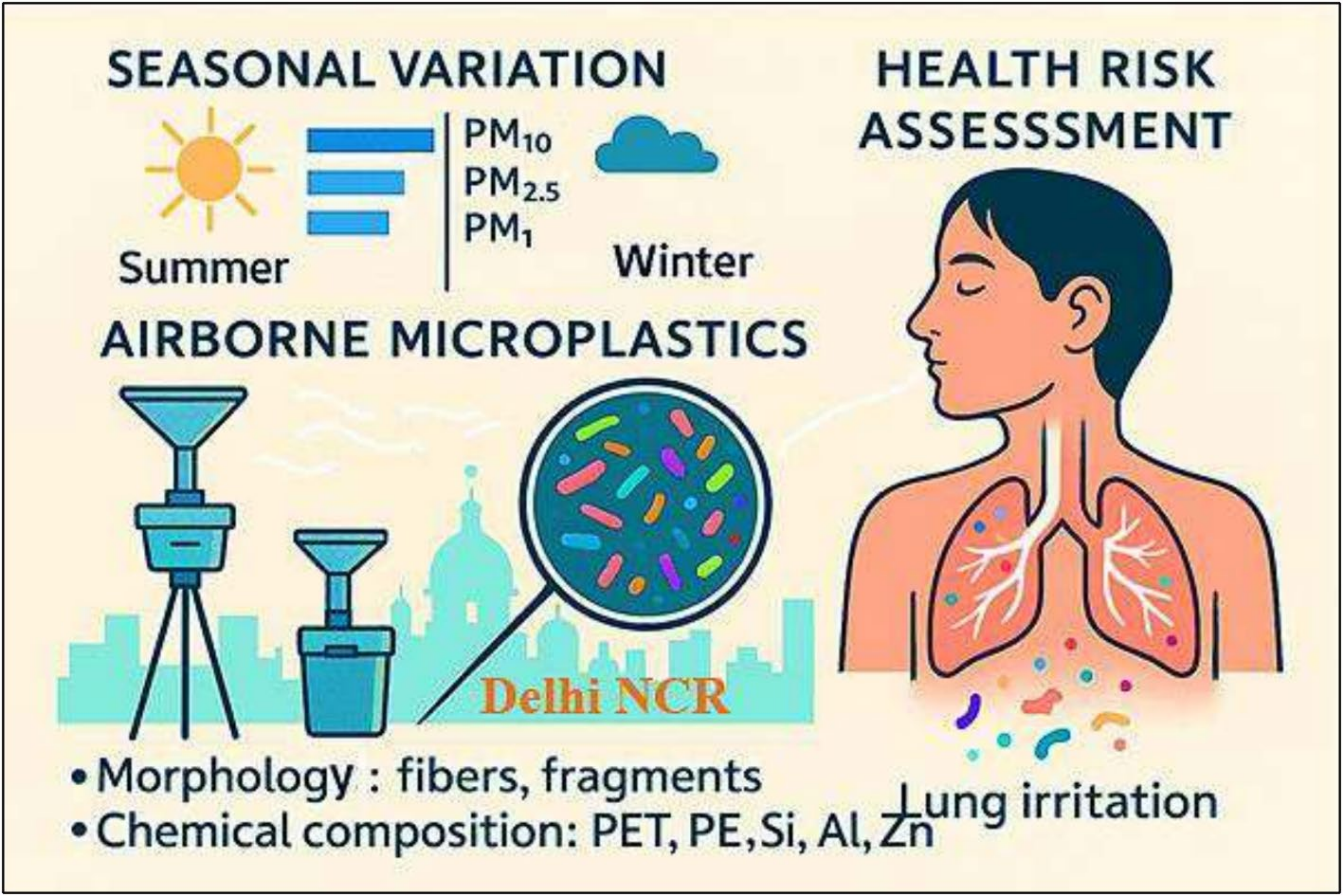
Overview of airborne MPs distribution, morphology, and potential health risk. The infographic illustrates the seasonal variation in MP concentrations in Delhi NCR, emphasizing higher levels during summer across PM₁₀, PM₂.₅, and PM₁ fractions. MPs identified were primarily fibers and fragments composed of PET and PE, along with trace metals (Si, Al, Zn). Inhalation of MPs may contribute to respiratory irritation due to particle deposition and associated contaminants.

This study aims to: (i) assess the extent of airborne MPs pollution at Lodi Road in Delhi, India; (ii) identify potential sources by analyzing their surface morphology and chemical composition; and (iii) evaluate the associated human health risks in an urban environment. A graphical summary of the study’s framework and findings is presented in Fig. 1. The results will offer critical insights into the characteristics of microplastic particles present in metropolitan air, contributing to a better understanding of their degradation, transport, and deposition processes. Furthermore, these findings will inform future research on the sources and health impacts of airborne MPs, supporting the development of targeted mitigation strategies and policies.

## Materials and methods

### Study area and sample collection

**Fig. 2:**
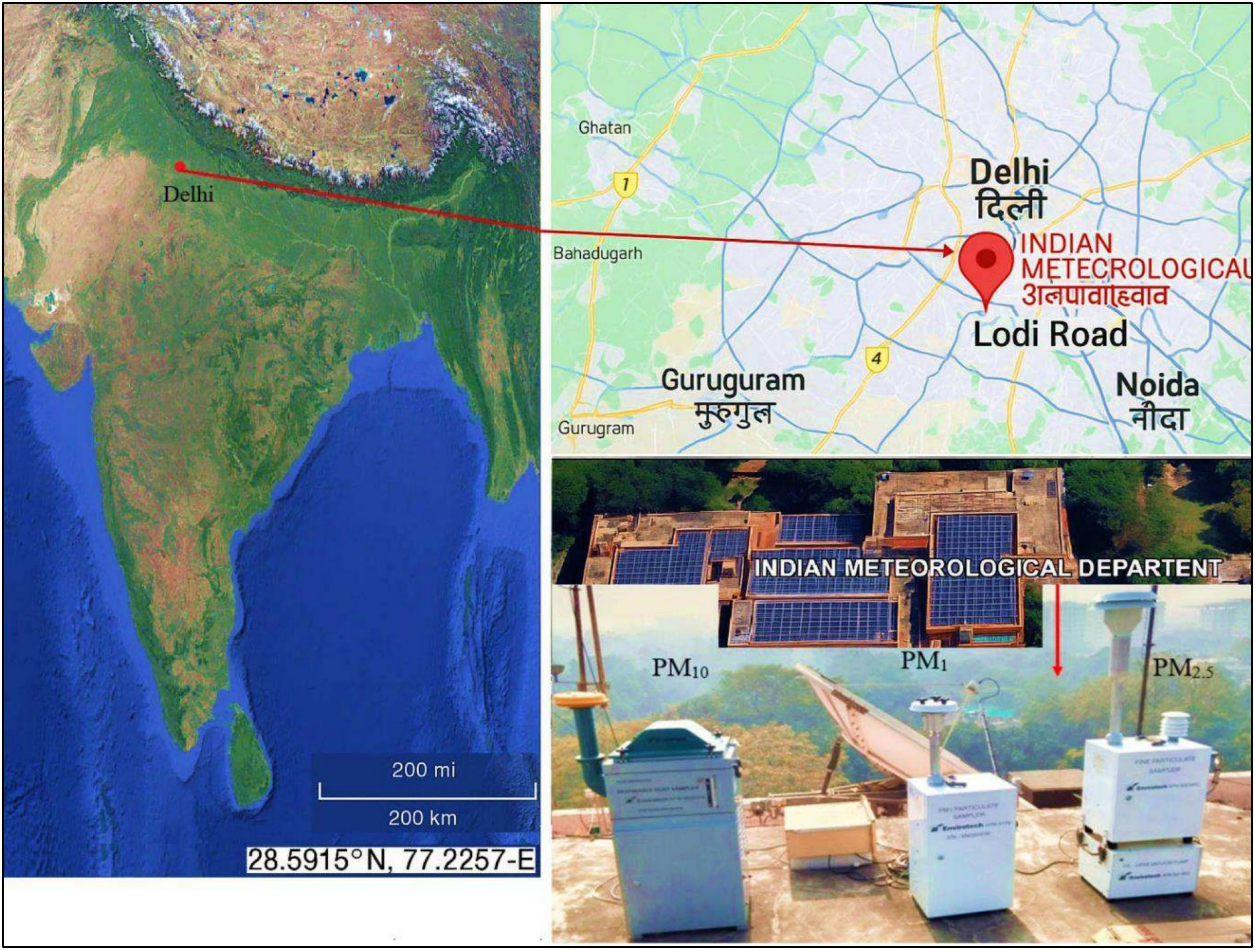
Location of the sampling site at Lodhi Road, Delhi (28.5915°N, 77.2257°E), India. Map and satellite imagery were generated using ArcGIS Pro (version 10.8; https://www.esri.com), using ESRI’s World Imagery Basemap.

In the present study, all measurements were carried out at the Lodi Road monitoring station, a central urban site in Delhi, situated in a high-traffic urban area with mixed commercial and institutional activity, was selected for its accessibility and relevance to typical urban exposure scenarios, the capital city of India (Fig. 2). Covering an area of approximately 1,483 square kilometers at an elevation of ~ 216 m, Delhi is home to a densely populated urban center with around 30 million residents. The city experiences extreme weather conditions, with summer temperatures reaching up to 45 °C and winter lows of around 5 °C, along with an average annual rainfall of approximately 750 mm. Outdoor microplastic (MP) samples associated with aerosols were collected using active pump samplers. These samplers segregate particles based on size, capturing them on filters by drawing air at a steady rate through specialized inlets designed for specific particulate sizes (PM_10_, PM_2.5_, and PM_1_). The sampling equipment was installed on the terrace of the Indian Meteorological Department (IMD) building in Delhi (28°58′N, 77°22′E), approximately 30 m above ground level. Sampling was conducted weekly, covering both weekdays and weekends per month (n = 4) of each particulate size sample to capture a representative dataset, using Quartz and polytetrafluoroethylene (PTFE) filters (47 mm diameter, pore size 0.5–2 µm) during the winter (January–March) and summer (April–June) seasons of 2024. These filters were chosen for their high thermal resistance, low organic background, chemical stability, low risk of contamination, and excellent efficiency in capturing fine particles. Low- and high-volume samplers were employed, specifically the PM_1_ sampler (Model Envirotech APM 577 M), PM_2.5_ sampler (Model Envirotech APM 550 MFC), and PM_10_ sampler (Model Envirotech APM 460 DXNL), operating at flow rates of ~ 10 m^3^/min, 16.7 m^3^/min, and 0.9–1.4 m^3^/min, respectively, over a 24-h period. After sampling, Quartz and PTFE filters were placed in a desiccator and equilibrated at 20–30 °C for the next 24 h. Filters were then wrapped in aluminium foil and stored at 4 °C until further analysis.

### Laboratory analysis: Visual inspection and quantification of MPs

Small sections (approximately one-quarter of a filter) were cut from each sample filter (100 cm^2^ for PM_10_ and 15 cm^2^ for PM_2.5_/PM_1_) using clean scissors. These pieces were placed in clean 100 mL glass beakers covered with aluminum foil, and all procedures were conducted in a contamination-free workspace. The samples were digested with 20% hydrogen peroxide (H₂O₂) for seven days at room temperature to remove organic matter. After digestion, the filters underwent 30 min of ultra-sonication, followed by rinsing with Milli-Q water to release adhered particles. The solution was further digested at 70 °C for one hour to ensure complete breakdown of residual organic material. The reaction was conducted in multiple stages, with fresh H₂O₂ added until the complete breakdown of visible organic debris was achieved. Care was taken to avoid excessive heat or agitation that might compromise the structural integrity of the MPs. Samples were filtered through 0.45 µm nitrocellulose filter papers and rinsed with Milli-Q water to remove H₂O₂. Density separation was achieved using a 1.2 g/cm^3^ NaCl solution, allowing MPs to float. The solution was shaken, settled, centrifuged at 4000 rpm, and vacuum-filtered three times. Finally, the samples were dried at 40 °C for 1–2 days. Blank samples underwent identical procedures.

MPs were identified and quantified based on visual characteristics (shape, color, and size) using a binocular microscope (OLYMPUS SZX16) with an auxiliary lens (SDF PLAPO 2 × PFC), offering a zoom range of 7 ×—115 × and up to 400 × magnification with WHN10/22 mm FOV eyepieces. MPs were identified based on specific criteria outlined by Hartmann et al. (2019)^16^, which included the following: 1) Fibers should have consistent thickness throughout and not be completely straight, without showing any cellular or natural structures; 2) Flat and thin pieces were categorized as films; 3) Round MPs were identified as spheres, while irregular or unclear shapes were classified as fragments. To detect smaller MPs that might be overlooked in traditional optical analysis, Nile Red (NR) staining was used. This method enhances the visibility of tiny MPs by highlighting how they interact with other materials^29,35^. A NR solution was prepared by dissolving 0.005 g of NR powder in 25 mL of acetone. Filter samples were stained with 2–3 drops of this solution and left for 24 h in a dark chamber at room temperature. NR dye, which fluoresces in response to environmental conditions, was used to stain MPs. A fluorescence microscope, with magnification up to 400 × and equipped with UV, RFP, and GFP filters (365/445 nm, 534/590 nm, and 450/565 nm), was employed to detect fluorescent MPs, including those smaller than 1 µm. MPs were classified by shape (fragments, fibers, films) and size (1–25 µm, 25–50 µm, 50–100 µm, 100–500 µm, 500–1000 µm). Common colors observed were transparent/white, blue, black, pink, yellow, and green. ImageJ software was used to measure the longest dimension of MPs from captured images.

### Surface morphology and chemical composition of MPs

To ensure accurate identification of MP particles prior to Scanning electron microscopy (SEM), a two-step confirmation process was followed. First, suspected MP particles were pre-identified using stereomicroscopy based on their color, shape, and non-biological appearance. These visually selected particles were then subjected to Fourier transform infrared spectrometer (FTIR) (Bruker) for polymer confirmation. FTIR spectra were acquired in the 600–4000 cm⁻^1^ range with an 8 cm⁻^1^ resolution, and spectral matches were compared against polymer reference libraries. Only those particles showing ≥ 85% match confidence to known polymer types (e.g., 1000 cm⁻^1^ for PS, 1720 cm⁻^1^ for PET, 1059 cm⁻^1^ for PE, and 695 cm⁻^1^ for PVC^36^ were classified as MPs and selected for further SEM analysis. These FTIR confirmed plastic particles were not chemically tagged but were physically isolated using metal tweezers and mounted onto carbon tape affixed to aluminum SEM stubs (~ 10 mm in diameter). The particles were then coated with a thin layer of platinum to improve conductivity. SEM (coupled with Energy-Dispersive X-ray Spectroscopy, (EDS); ULTIM MAX 40, Oxford Instruments) was used to examine surface features such as cracks, fractures, and pits and to assess elemental composition of the MPs was assessed using EDX detector. To evaluate the method recovery efficiency, spike-recovery experiments were conducted. Known concentrations of standard particles containing Si, Al, and Zn were spiked onto blank filters and subjected to the full digestion and analysis workflow. The average recovery rates were found to be Si (88.4%), Al (91.2%), and Zn (89.5%), confirming the method’s ability to retain relevant particles and associated elements with high reproducibility. These results strengthen the methodological reliability and validate the analytical techniques employed in the study.

### Health risk assessment

The estimated inhalation exposure to MPs on both normal and dusty days was calculated following the guidelines provided by the World Health Organization (WHO, 2000)^37^. The average daily intake (ADI) was determined using the formula:

$${\text{ADI }} = {\text{ C }} \times {\text{ IR}}$$

where C represents the concentration of MPs (in MPs/m^3^) and IR is the age-specific inhalation rate (in m^3^/day). For this assessment, the population was categorized into five age groups: infants (≤ 1 year), toddlers (1–6 years), children (6–12 years), adolescents (12–18 years), and adults (≥ 18 years). The corresponding inhalation rates (IR) considered for these groups were 5.4, 9.0, 12.0, 15.7, and 15.7 m^3^/day, respectively^29^.

### Experimental quality control

To prevent potential contamination of samples during both the field and lab work. Before using, glass petri dishes for collecting samples and storage containers were thoroughly cleaned with diluted hydrochloric acid followed by Milli-Q water. Further dried and covered in aluminum foil, which was removed only when taking samples. To carry out the extraction processes were done in a clean airflow area to avoid contamination from airborne materials after collection. All procedures, cotton lab coats and single use nitrile gloves were worn during the lab work. A combination of three procedural blanks during sample processing to prevent airborne MPs contamination from outside sources. Checking the blank samples showed that there were no MPs in these samples.

## Results and discussion

### The abundance of MPs

**Fig. 3:**
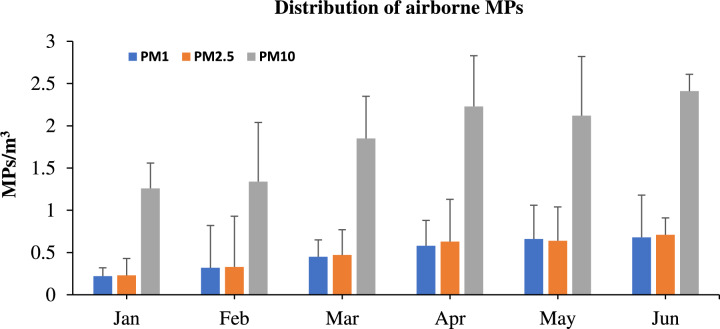
Distribution of MPs in all particulate factions (PM10, PM2.5 and PM1) during the study period.

A total of 2,087 MPs were identified in the air across all particulate samples during the study period in Delhi. The concentration of MPs ranged from detected MPs varied between 1.26–2.41 MPs/m^3^ (mean: 1.87 ± 0.5 MPs/m^3^) in PM_10_, 0.23–0.71 MPs/m^3^ (mean: 0.51 ± 0.2 MPs/m^3^) in PM_2.5_, and 0.22–0.68 MPs/m^3^ (mean: 0.49 ± 0.2 MPs/m^3^) in PM_1_, with significant variations (t-test, p < 0.05) observed throughout the study period (Fig. 3). The highest concentrations were found in PM_10_, followed by PM_2.5_ and PM_1_. The presence of airborne MPs in the study area may be attributed to the high population density in Delhi, the capital city of India, which is characterized by busy markets, schools, manufacturing plants, and intense daily human activity. MPs likely originate from nearby plastic waste and trash discarded in open spaces. Meteorological conditions significantly influence the movement and dispersion of MPs across the urban landscape, with winds potentially transporting them from surrounding regions into the city. Additionally, meteorological conditions play a crucial role in the movement and distribution of MPs throughout the city. Winds could also carry MPs from surrounding areas into Delhi. To contextualize the findings of this study, airborne MP concentrations were compared with regional, national, and international datasets (Table 1). The observed MP concentrations in Delhi NCR (PM₁₀: 1.87 ± 0.5 MPs/m^3^; PM₂.₅: 0.51 ± 0.2 MPs/m^3^; PM₁: 0.49 ± 0.2 MPs/m^3^) are comparable to those reported in coastal India (Arabian Sea: 1.30–1.46 MPs/m^3^)^2^ and California (3.3 MPs/m^3^)^38^, but lower than values observed in certain indoor environments such as Turkey (12.03–18.51 MPs/m^3^)^9^. Urban outdoor concentrations reported from Paris (0.3–1.5 MPs/m^3^)^31^ and Mexico City (0.2–0.1 MPs/m^3^)^20^ were lower than those observed in Delhi. Variations across studies can be attributed to differences in sampling techniques (active vs. passive), particle size detection limits, environmental settings (indoor vs. outdoor), and local plastic use patterns. Moreover, climatic factors such as wind speed, humidity, and atmospheric stability influence MP dispersion and deposition dynamics. Overall, the findings position Delhi NCR within the global range of reported airborne MP levels, emphasizing the significance of MPs as an emerging component of urban air pollution.

**Table 1:** Comparison of airborne MPs concentrations across different regions.

Study location	/m^3^)	PM₁₀ (items PM₂.₅ (items/m^3^)	PM₁ (items/m^3^)	Sampling method	Dominant polymer types	References
Delhi NCR, India (This study)	1.87 ± 0.5	0.51 ± 0.2	0.49 ± 0.2	Active (PM₁₀/PM₂.₅/PM₁ samplers)	PET, PE, PP, PS, PVC	This study
Arabian Sea (India, Goa)	1.30–1.46	-	-	High volume TSP sampler	PVC, PMMA, Polyester (PES), SBMA, POM	^39^
California, USA	3.3 ± 1.9	-	-	Active sampler	PS, PET, PE, PVC, PC, PA, ABS	^40^
Bushehr Port, Iran	5.2	5.2	-	Street dust/suspended particles	PE, PP, PS	^29^
Paris, France	0.3–1.5	-	-	Atmospheric deposition (passive)	PE, PP, PS, PET	^31^
Mexico City, Mexico	0.2	0.1	-	Urban dust/air	PE, PET	^20^
Spain	1.5–13.9	-	-	Suspended atmospheric MPs	PA, PU, PES, PE, PS	^41^
Sri Lanka	0.1–0.9	-	-	Outdoor ambient air	PET, PES, Acrylic, PA, PP, PS	^42^
China (Multiple Cities)	0–4.18	-	-	Suspended atmospheric MPs	PET, PE, PES, PAN, PAA	^43^
Turkey (Indoor)	12.03– 18.51	-	-	Indoor air (active sampling)	PA6, PVC, PP, PS, HDPE	^9^

The monthly distribution of detected airborne MPs across all particulate fractions showed a gradual increase from January to June, with the highest concentrations observed in June and the lowest in PM_1_ during January. The observed anomaly in May, where the concentration of MPs in PM₂.₅ is lower than in PM₁, may be explained by several factors including sampling variability, local source contributions, and aerosol dynamics under specific meteorological conditions. One possibility is the presence of fine and ultrafine microplastic particles originating from secondary fragmentation, combustion processes, or abrasion of synthetic materials, which tend to fall within the PM₁ size range^41^. Such particles may bypass coarser size fractions and accumulate in PM₁ due to their small size and low settling velocity. Additionally, resuspension of ultrafine particles from indoor or traffic related sources may have contributed to the elevated PM₁ concentrations. Environmental factors such as increased solar radiation and temperature during May could also enhance photodegradation of plastics and promote the formation of finer fragments^42^. Similar findings, where higher MP counts were reported in finer fractions than expected, have been documented in other air quality studies and are often attributed to combustion emissions, urban activities, and aerosol transformation processes^43^. While this trend was not consistent across all months, it highlights the complexity of microplastic size distribution in ambient air and the need for further research to better understand particle behavior under varying environmental conditions. The average number of MPs found in the study area was higher during the summer season compared to the winter season for all particulate fractions. During the summer season, the average concentration in PM_10_ was 2.24 ± 0.15 MPs/m^3^, while in the winter season, it was 1.48 ± 0.32 MPs/m^3^. For PM_2.5_, the average concentration was 0.66 ± 0.1 MPs/m^3^ in the summer season and 0.34 ± 0.12 MPs/m^3^ in the winter. In PM_1_, the average was 0.64 ± 0.1 MPs/m^3^ in the summer season and 0.33 ± 0.1 MPs/m^3^ in the winter. The concentration of MPs in PM_10_ and PM_2.5_ decreased from June to January in the study area. The number of MPs varies with the seasons, influenced by weather conditions. Significant variations (t-test, p < 0.05) in temperature and winds across seasons strongly impact the distribution and concentration of MPs in Delhi. Higher MP concentrations during the summer season are likely due to intense sunlight and elevated temperatures, which degrade materials and release smaller plastic particles into the environment. This indicates that MPs may further degrade and disperse over greater distance. The higher concentration of MPs during the summer season can be attributed to the strong sunlight and high temperatures, which break down materials and release tiny plastic particles into the environment. This suggests that MPs may degrade further and spread over longer distances. A similar pattern was observed in an earlier study conducted in Mexico City^20^.

### Physical characteristics of MPs

**Fig. 4:**
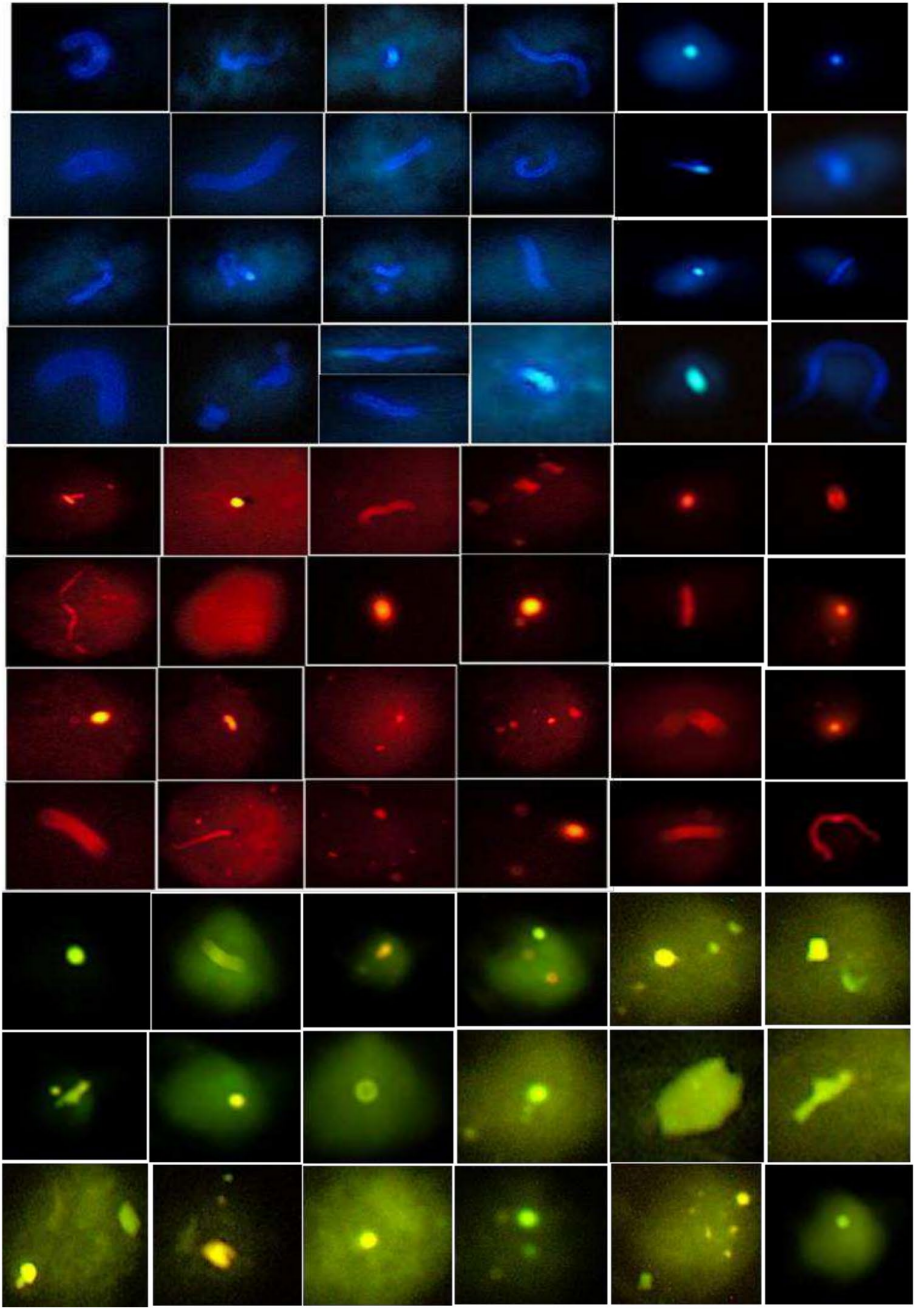
Photographs under a fluoresce microscope with blue, red, and green color showing airborne MPs in the study area.

**Fig. 5:**
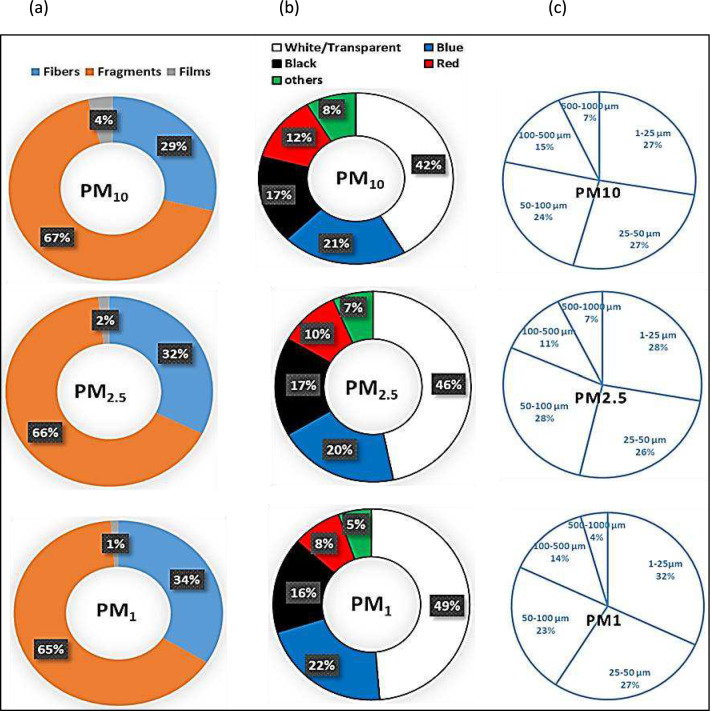
Percentage distribution of airborne MPs in different particulate factions (**a**). shape (**b**). color (**c**). size.

In this study, fluorescence microscopy revealed that MP particles were predominantly fragments and fibers across all PM size fractions (PM₁₀, PM₂.₅, and PM₁) during the sampling period in Delhi (Fig. 4). Among the identified types, fragments were the most frequently observed, whereas films were the least common. Fragments and fibers accounted for 67% and 29% of MPs in PM_10_, 66% and 32% in PM_2.5_, and 65% and 34% in PM_1_, respectively (Fig. 5a). Fragments and fibers made up 67% and 29% of the total MPs in PM_10_, 66% and 32% in PM_2.5_, and 65% and 34% in PM_1_, respectively. The distribution of MPs in different shapes remained consistent throughout the months. It is important to note that identifying MPs smaller than 1 µm was challenging but using a fluorescence microscope with up to 400 × magnification facilitated their identification. Fluorescence analysis is a reliable and straightforward method for detecting and quantifying MPs in environmental samples^29^. In this study, visible, green, and blue lights were used for fluorescence, as MPs glowed strongly in blue, red, and green, while non-plastic particles did not exhibit any fluorescence. After treatment with hydrogen peroxide (H_2_O_2_) and Nile Red dye, natural contaminants did not glow^44^, and rubber pieces did not fluoresce under ultraviolet light^45^.

Human activities in Delhi, a densely populated capital city, significantly contribute to the generation of various types of MP particles, particularly from industrial sources. Similarly, metropolitan areas often produce many small plastic pieces (MPs) that can travel long distances through the air and wind, eventually mixing with dust and particulate matter in the atmosphere^46^. The microplastic fragments likely originate from both primary (e.g. industrial processes and personal care products), and secondary sources, with plastic waste degradation being the primary cause of small plastic pieces. This occurs as plastic waste, such as bags, wrappers, and packaging, breaks down into smaller fragments in urban environments due to physical degradation and abrasion. Similar findings have been reported in previous studies, which also observed significant amounts of tiny plastic pieces in air samples^20^. Plastic fibers in cities typically come from sources like clothing, cars, ropes, carpets, and laundry. Washing and drying clothes is a significant contributor to the release of tiny plastic fibers into the environment. Which indicates that indoor environments often exhibit higher MP concentrations, and thus indoor exposure may be more significant^9^. Additionally, the MPs were categorized by color^47^. The colors of the detected MPs in all particulate samples were primarily transparent/white (45.7%), blue (21.0%), black (16.7%), red (10.0%), and other colors (6.7%) (Fig. 5b). White/transparent MPs were found in large quantities during both seasons in the study area. There was little variation in the color distribution of MPs across PM_10_, PM_2.5_, and PM_1_ samples. The high prevalence of white MPs is likely due to items such as plastic bags, packaging, wraps, containers, and degraded plastic materials. The red and brown plastic fibers found in this study were mostly attributed to washing clothes in urban areas. The size of airborne MPs is crucial for understanding their impact on pollution and their potential effects on the environment and human health. In this study, airborne MPs were found to range from 1 µm to 1000 µm across all particulate matter (PM) samples (Fig. 5c). The smallest size observed was in fragments (1–10 µm), while the largest was in fibers (10–1000 µm). The results indicated that most of the particles were smaller < 25 µm, with fewer larger MPs, suggesting that larger MPs may have broken down into smaller fragments due to weathering processes. The range of microplastic sizes found in this study was broader than what the air samplers could measure (PM_10_, PM_2.5_, and PM_1_). This discrepancy may be attributed to the differing aerodynamic behaviors of MPs compared to conventional dust particles, as well as their orientation and position relative to the sampler inlet during collection. While the size distribution suggests the presence of even smaller plastic particles (less than 1 µm), our study was unable to detect them due to the lower size limit of the measurement equipment.

### Plastic chemical composition and identification of sources

**Fig. 6:**
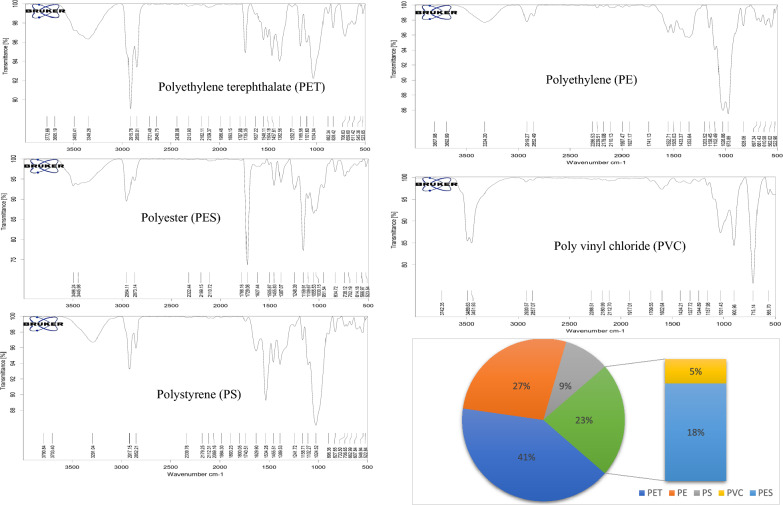
Major identified polymers of collected airborne MPs in the study area.

The chemical composition of the detected MPs was analyzed using FTIR, which revealed five major polymer types across all PM samples during the study period (Fig. 6). The identified MPs included PET (41%), PE (27%), PES (18%), PS (9%), and PVC (5%). These findings confirm the presence of airborne MPs originating from common urban sources. PET was the most abundant polymer, followed by PE. Characteristic FTIR spectral features included C–H stretching vibrations (~ 2920–2850 cm⁻^1^), C–H₂ bending (~ 1430–1350 cm⁻^1^), C = O stretching (~ 1729 cm⁻^1^), C–O stretching (~ 1250–1030 cm⁻^1^), aromatic ring vibrations (~ 1620–1380 cm⁻^1^), and C–H₂ rocking modes (~ 880–705 cm⁻^1^), indicating the presence of these polymers. These plastics are widely used in packaging, single-use items, reusable bags, beverage bottles, and textiles. The predominance of PET and PE may be attributed to the high volume of plastic waste generated in Delhi, which produces approximately 690 tons of plastic waste daily due to its dense population and rapid industrialization. According to CPCB (2022), of the 11,335 tonnes per day (TPD) of municipal solid waste generated in Delhi, approximately 1,145 TPD is plastic waste, with single-use plastics contributing about 635 TPD. PVC was identified by its characteristic C–Cl stretching vibration around ~ 700 cm⁻^1^ and is commonly used in packaging materials, wall and floor coverings, electrical wires, automobile components, and furniture. PES was recognized through its spectral bands between 1248–951 cm⁻^1^, associated with ester group vibrations (C–O), aromatic C = C bands, symmetrical C–O–C stretching, and a C = O band at 1729 cm⁻^1^. This polymer is predominantly found in synthetic fabrics and is released through the wear and laundering of clothing.

The dominance of PET/PES fibers and PE fragments in the airborne MPs can be attributed to multiple local sources and emission pathways common in densely populated urban-industrial settings. PES and PET fibers are widely released from textile manufacturing units, garment processing industries, and domestic laundry activities, which are prevalent in and around the Delhi NCR region. Fine synthetic fibers are easily shed during washing and drying of clothes and can become airborne through ventilation systems or open drying practices. In parallel, the widespread use and poor disposal of PE based packaging materials, plastic bags, and consumer products contribute to the presence of PE fragments. Open burning of municipal solid waste, a common practice in many peri-urban and informal settlements, also serves as a significant source of fragmented MPs, especially PE and other low-density polymers, released during incomplete combustion. Likewise, vehicular wear-and-tear, abrasion of road markings, and secondary fragmentation from construction debris and degraded urban plastic waste further contribute to airborne MP loads. These diverse sources and pathways highlight the complex urban emission matrix responsible for MPs pollution in ambient air. Additionally, wind rose diagrams were generated to illustrate the distribution of wind directions and wind speed ranges at the sampling site in Delhi during the entire study period (Fig. S1). The analysis indicates a predominant wind flow mostly from the northwest sector, suggesting that this direction may serve as a potential transport pathway for airborne MPs to the study location. The elevated MP concentrations observed in Delhi are likely influenced by regional anthropogenic activities, combined with prevailing north-westerly winds that facilitate the long-range or regional-scale transport of plastic particles.

### Surface morphology and elemental composition of MPs

**Fig. 7:**
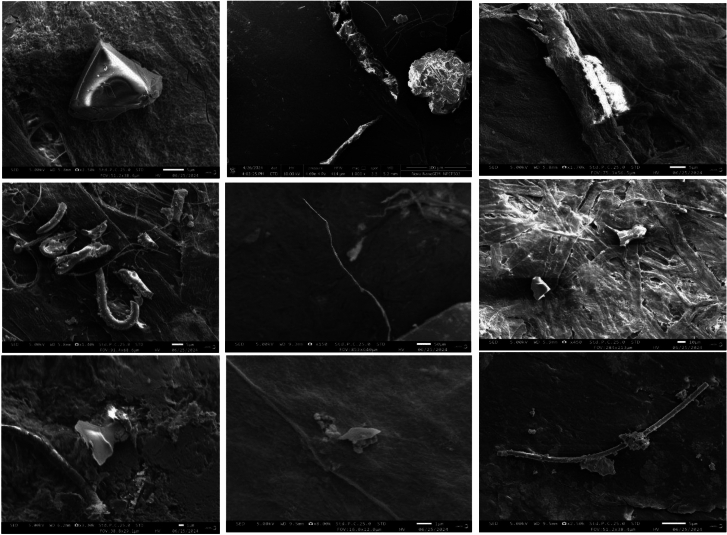
Few images of SEM showing different morphology (straight breaks, splits, and smooth edges) of collected airborne MPs.

**Fig. 8:**
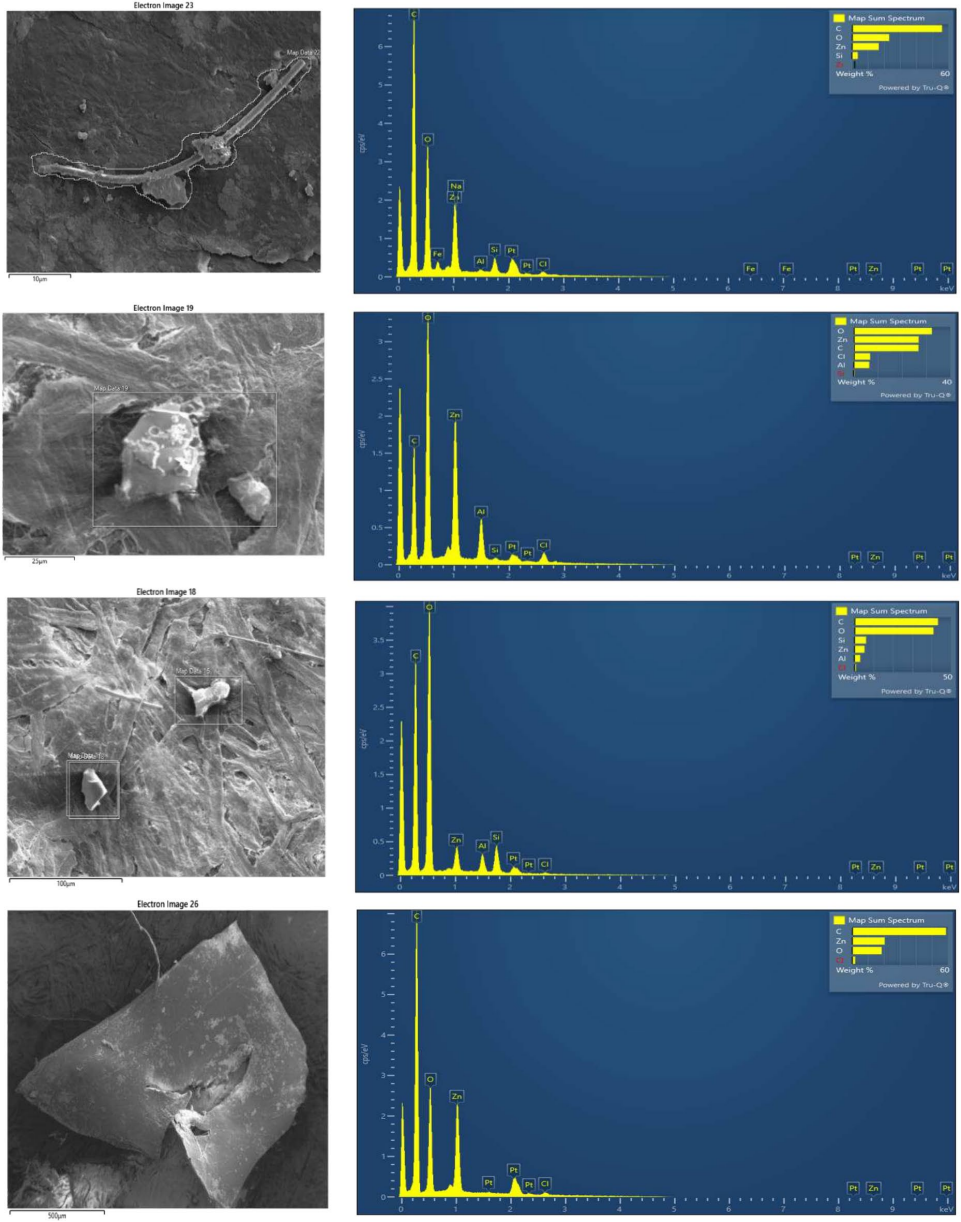
Elemental composition of collected airborne MPs by SEM–EDS.

To examine the surface morphology and elemental composition of MPs, SEM–EDS was employed. The surface morphology was predominantly characterized by homogenous and and degraded pieces (Fig. 7), exhibiting features such as straight breaks, splits, and smooth edges. These characteristics suggest that the MPs have been exposed to the elements and weathered over time. Smooth edges indicate that the particles are newer to the environment, while split edges suggest they have been present longer and have undergone physical degradation^43,48^. Some MPs also showed evidence of other small particles adhering to their surfaces, implying that airborne MPs can collect additional particles carried by the wind. This combination of particles makes the MPs even more hazardous to human health when inhaled^17^. Elemental analysis using EDS (Fig. 8) confirmed that MPs were primarily composed of carbon (C) and oxygen (O), along with trace amounts of inorganic elements such as Zn, Si, Al, and chlorine (Cl), These elements may be either additives in the plastic or environmental adsorbates. The presence of such elements is consistent with prior studies on airborne MPs^27^. Metals and organometallic compounds are commonly used as fillers, stabilizers, colorants, or additives in plastic production. For example, Si, Al, and calcium (Ca) are often incorporated into polymers to enhance functionality and reduce manufacturing costs^49,50^. Inorganic elements may also be adsorbed from the surrounding environment, either during production or after atmospheric exposure. To improve our understanding of seasonal differences in MP composition, future research should focus on conducting elemental mapping of air samples collected during both winter and summer seasons. This analysis would provide valuable information on the elemental makeup of MPs, allowing for a clearer assessment of their chemical characteristics and possible sources. Comparing elemental profiles across seasons could highlight variations in MP types, source contributions, and potential interactions with other airborne pollutants under differing climatic conditions. Incorporating this approach would strengthen the link between seasonal atmospheric changes, emission sources, and the prevalence of specific MP particles in urban environments, ultimately contributing to a more comprehensive understanding of their distribution and behavior.

### Human health risk assessment

**Fig. 9:**
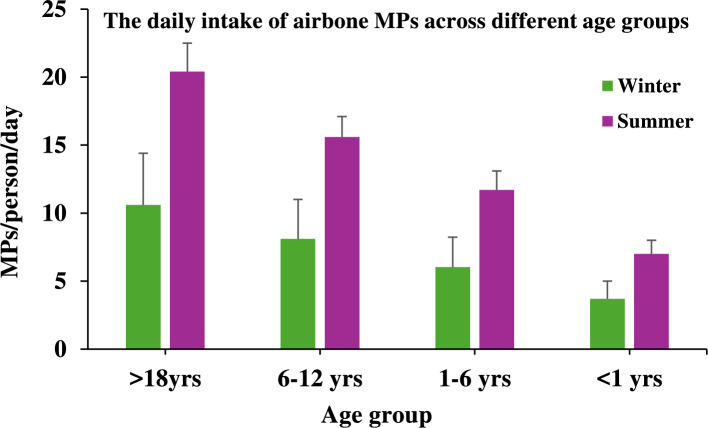
Average daily intake of airborne MPs across different age groups (> 18 years, 6–12 years, 1–6 years, and < 1 year) during winter and summer seasons.

The risk assessment of airborne MPs was based on an average daily air intake of 10–20 m^3^ for adults^51,52^. These fine particles can enter the human respiratory system through inhalation, potentially causing adverse health effects such as respiratory irritation and lung damage^43^. The average daily intake of airborne MPs varied significantly across different age groups and seasons, with a ~ 90% confidential interval (Fig. 9). In general, higher inhalation exposures were observed during the summer compared to the winter season across all age categories. For adults (> 18 years), the average daily intake was approximately 10.7 ± 3.8 MPs/person/day during winter, increasing to 21.1 ± 2.1 MPs/person/day in summer. Similarly, children aged 6–12 years exhibited an intake of 8.1 ± 2.9 MPs/person/day in winter, rising to 15.6 ± 2.0 MPs/person/day during summer. Younger children aged 1–6 years inhaled approximately 6.1 ± 2.2 MPs/person/day in winter and 11.7 ± 2.0 MPs/person/day in summer. Infants (< 1 year) had the lowest exposure, with an estimated intake of 3.6 ± 1.3 MPs/person/day in winter and 6.8 ± 1.4 MPs/person/day in summer. The seasonal variation may be attributed to differences in meteorological conditions, particularly reduced precipitation and increased dust resuspension during summer, which enhances airborne particle concentrations. Higher temperatures and lower humidity in summer may also facilitate the fragmentation and resuspension of MPs into the atmosphere. Among the age groups, adults showed the highest intake rates, likely due to greater daily inhalation volumes and outdoor activities. However, the relative health risk could be more significant for younger children and infants, given their developing respiratory systems, higher breathing rates relative to body weight, and greater physiological vulnerability. Moreover, the higher intake rates observed during the summer season underscore the potential for increased health risks during drier periods, particularly for vulnerable populations such as children and infants. These results highlight the urgent need for further toxicological studies to evaluate the health impacts of chronic exposure to airborne MPs, particularly under varying seasonal and demographic conditions.

The estimated inhalation exposure to airborne MPs in Delhi NCR (5.3–15.4 MPs/day, corresponding to approximately 1,935–5,621 MPs annually) was found to be higher than reported levels in Mexico City (2.4 MPs/day, approximately 876 MPs annually)^20^ but lower than indoor exposure estimates from Scotland (38–187 MPs/day, approximately 13,731–68,415 MPs annually)^53^. This pattern aligns with previous findings that indoor environments typically exhibit higher MP concentrations due to limited air exchange and accumulation of synthetic fibers from indoor sources. Comparatively, outdoor urban air in Paris^31^ and Sri Lanka^39^ exhibited lower daily exposure estimates, reflecting regional differences in plastic use, urban density, sampling techniques, and meteorological influences. These comparisons highlight that while Delhi’s outdoor MP exposure is moderate on a global scale, it remains a significant public health concern, particularly given the city’s high baseline levels of PM pollution. Although no threshold exists for safe MP inhalation, chronic exposure particularly in occupational settings may pose health risks including asthma, bronchitis, pneumonia, lung inflammation, and cancer^32,54^. Smaller MPs may carry bacteria and induce oxidative stress, potentially affecting not just lung function but also skin and brain cells^55^.Inhalation is not the only exposure route. MPs may contaminate food or be ingested during routine activities in polluted environments. Exposure levels vary by age, sex, occupation, inhalation rate, and health status. Wind can transport MPs over long distances, extending their environmental impact^29^. Health risks may be amplified by co-exposure to other airborne pollutants like heavy metals and POPs^56,57^. MPs act as vectors for toxic substances including PM₂.₅, lead and cadmium due to their large surface area and hydrophobic nature^58^. These compound laden MPs can penetrate deep into the lungs, triggering inflammation, oxidative stress, and immune responses, and potentially reaching the bloodstream or nervous system^59^. Additionally, MPs may disrupt epithelial barriers and promote translocation of toxic substances into systemic circulation, exacerbating potential cardiovascular or neurological impacts. Daily MP intake estimates are subject to uncertainty due to individual physiology, environmental variability, and methodological assumptions. Inhalation volumes vary with age and activity, ranging from 6–20 m^3^/day^60^. Not all inhaled MPs are retained; deposition depends on particle size, shape, and density. Fibers tend to settle in upper airways, while ultrafine particles (< 1 µm) may reach alveoli^61^. MP concentrations also fluctuate seasonally and with proximity to emission sources. Estimating MP dose by count involves assumptions about particle density and shape, which introduces further uncertainty^62^. Hence, current estimates serve as approximations, and future studies should adopt real-time monitoring, individualized exposure models, and improved analytical methods to enhance accuracy^63^.

### Limitations and recommendations

The analytical approach used in this study primarily optical microscopy limits our ability to detect MPs smaller than 1 µm, particularly nanoplastics (NPs). This methodological constraint means that ultrafine plastic particles, which may have significantly different physical and toxicological properties compared to larger MPs, were likely underrepresented or undetected in our samples. The exclusion of particles < 1 µm could result in a substantial underestimation of potential health risks, particularly from the inhalation exposure pathway. NPs can penetrate deeper into the respiratory system, reaching the alveolar regions, and due to their high surface area-to-volume ratio, they may adsorb and transport toxic chemicals more effectively than larger particles. Moreover, NPs have been shown to cross biological barriers, such as the alveolar capillary membrane, potentially entering the systemic circulation and reaching vital organs. These properties suggest that NPs may pose higher toxicity than larger MPs due to their small size, greater reactivity, and enhanced cellular uptake. Future research employing higher-resolution techniques such as electron microscopy or Raman spectroscopy with submicron detection capability is essential for a more accurate risk assessment.

Based on the observed prevalence of airborne MPs, particularly PET/PES fibers and PE fragments, several actionable interventions can be recommended to mitigate emissions and reduce human exposure. Strengthening waste management infrastructure is critical specifically targeting the reduction of open waste burning, improving segregation at source, and ensuring safe disposal or recycling of plastic materials. Regulations limiting uncontrolled dumping and incentivizing the adoption of biodegradable alternatives can also help curb plastic pollution at the source. In the textile sector, the implementation of filtration systems in washing facilities and industrial laundries could significantly reduce fiber emissions into the air. Additionally, raising public awareness about the environmental and health implications of MPs such as through community outreach and educational campaigns can foster behavioral change, including responsible plastic use and disposal practices. Policymakers and urban planners should also consider integrating MP monitoring into existing air quality management frameworks to inform data-driven interventions. The growing evidence of airborne MPs in urban environments and their potential to impact human health underscores the urgent need for regulatory frameworks specifically targeting MP pollution in air. Unlike well-established air quality standards for particulate matter (e.g., PM₂.₅ and PM₁₀), there are currently no guidelines or threshold limits for MPs, despite their ability to persist in the atmosphere and interact with co-pollutants. Given their diverse morphology, polymer composition, and capacity to adsorb toxic substances, airborne MPs may pose unique health risks not fully captured by existing PM metrics. Establishing regulatory standards for airborne MPs would help guide monitoring efforts, inform risk assessments, and drive mitigation strategies like how PM₂.₅ regulations have shaped air pollution control policies. Furthermore, the development of standardized sampling and analytical protocols is crucial for generating comparable datasets that can support the formulation of evidence-based regulatory thresholds. Such proactive regulatory approaches are essential to address this emerging pollutant and protect public health.

## Conclusions

This study presents one of the first detailed investigations into the occurrence, characteristics, and inhalation risks of airborne MPs in the Delhi NCR region, India’s capital. Our findings reveal that MPs are a persistent and seasonally variable component of urban air, with a predominance of fragment and fiber particles composed mainly of PE and PET. The elevated concentrations during the summer season, along with evidence of long-range transport via prevailing winds, highlight the complex interplay between local emissions, atmospheric dynamics, and urban MP pollution. Estimated daily inhalation exposure levels, particularly among sensitive populations such as children and infants, raise legitimate concerns regarding long-term health outcomes. While definitive toxicological thresholds for airborne MPs have yet to be established, their ability to adsorb and transport hazardous contaminants suggests that even low-level exposure could contribute to cumulative respiratory and systemic health burdens. The study highlights the urgent need for the inclusion of MPs in air quality monitoring frameworks and environmental health risk assessments. Future research should focus on identifying ultrafine and nano-plastic particles, understanding synergistic effects with other airborne pollutants, and establishing dose–response relationships. Moreover, long-term, multi-site monitoring is essential to evaluate spatial variability and better assess the broader public health implications of airborne MP exposure. Considering these findings, the integration of MPs into urban air quality management and pollution mitigation strategies is imperative for safeguarding environmental and human health.

## Supplementary Information

Supplementary Information.

## Data Availability

All data supporting the findings of this study are included within the manuscript.
