# Correction: Characterization and health risk assessment of airborne microplastics in Delhi NCR

**DOI:** 10.1038/s41598-025-16583-4

**Published:** 2025-08-26

**Authors:** Molla Nageswar Rao, Sachin D. Ghude, Sandip S. Nivdange, Rajani Panchang, Atar Singh Pipal, Arkabanee Mukherjee, Hardeep Sharma, Vikash Kumar

**Affiliations:** 1 Ministry of Earth Sciences, Indian Institute of Tropical Meteorology, Pune, 411008 India; 2 Department of Environmental Sciences, Savitribai Phule Pune University, Pune, 411007 India

Correction to: *Scientific Reports* 10.1038/s41598-025-04306-8, published online 15 July 2025

The original version of this Article contained an error in the spelling of the author Sachin D. Ghude which was incorrectly given as Sachin S. Ghude.

Additionally, in the author contributions section:

“Sachin S. Ghude: Conceptualization, Validation, Writing, Review & Editing, Supervision, Project administration;”

Now reads:

“Sachin D. Ghude: Conceptualization, Validation, Writing, Review & Editing, Supervision, Project administration;”

The original Article has been corrected.

