# Supplementary material for: Characterization and health risk assessment of airborne microplastics in Delhi NCR
Source: Sci Rep. 2025 Jul 15;15:25662. doi: 10.1038/s41598-025-04306-8 (PMC12263988; doi:10.1038/s41598-025-04306-8)
Supplement: Supplementary file 1 — Supplementary Information. [file 41598_2025_4306_MOESM1_ESM.pdf]

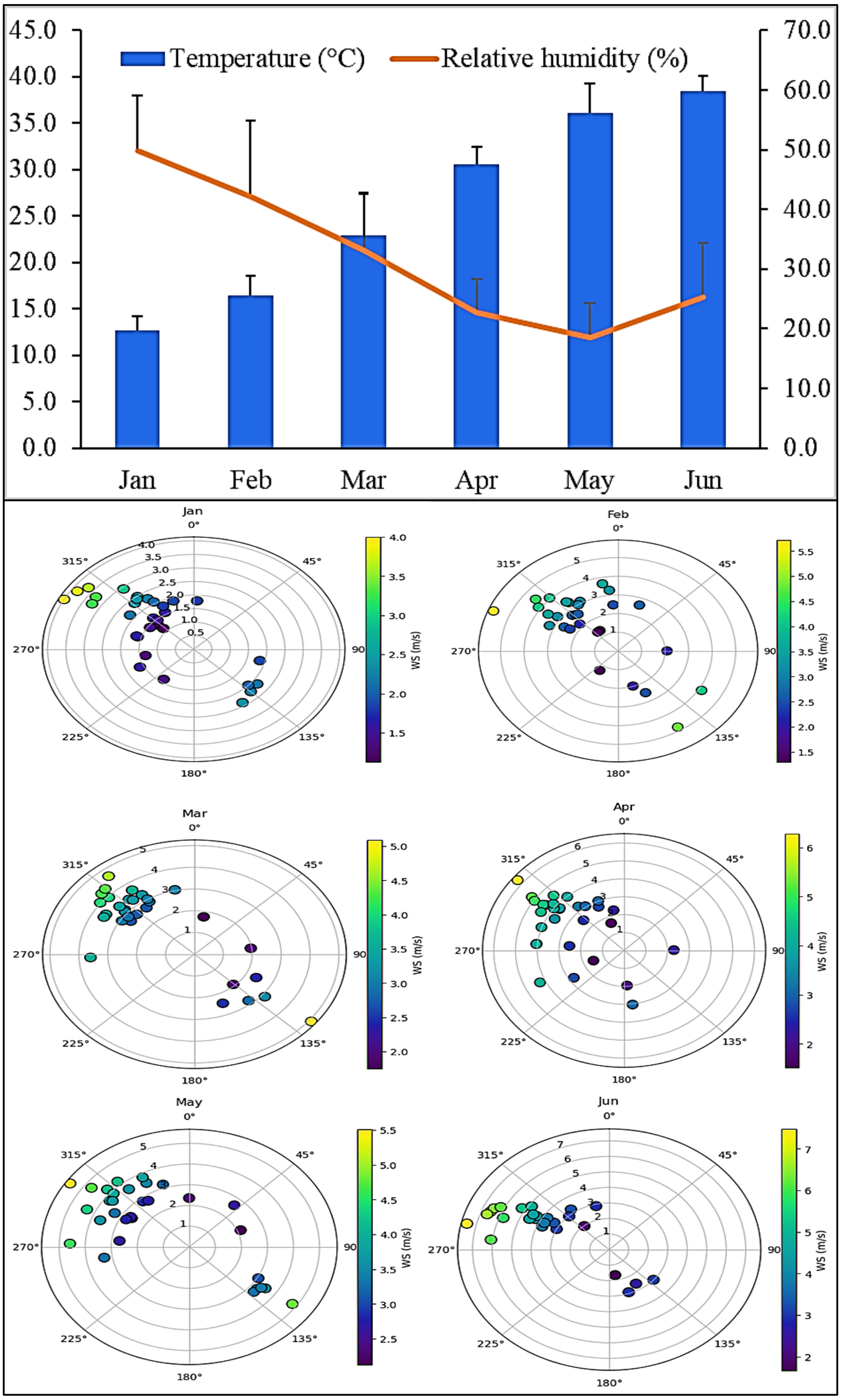

Fig. S1. Monthly meteorological conditions, including temperature, relative humidity, and wind rose plots illustrating wind speed and direction patterns, were analysed at the study site in Delhi. Whereas the data of meteorological parameters were collected from satellite measurements (https://power.larc.nasa.gov/data-access-viewer/).
